# Seeing the person before the teeth: A realist evaluation of a dental anxiety service in Norway

**DOI:** 10.1111/eos.12860

**Published:** 2022-02-26

**Authors:** Emilie Bryne, Sarah Catherine Patricia Duff Hean, Kjersti Berge Evensen, Vibeke Hervik Bull

**Affiliations:** ^1^ Oral Health Centre of Expertise Stavanger Rogaland Norway; ^2^ Faculty of Social Sciences at the University of Stavanger, Stavanger Norway

**Keywords:** abuse, cognitive behavioural therapy, dental phobia, person‐centred care, torture

## Abstract

Patients with a trauma history, whether sexual abuse or torture, or dental phobia, tend to avoid dental services due to severe dental anxiety. Subsequently, they experience poor oral health, lower quality of life, and poorer general health. In Norway, a specific service (torture, abuse, and dental anxiety [TADA]) targets these patients’ dental anxiety through cognitive behavioural therapy (CBT) prior to dental restoration. By exploring patients’ experiences with TADA services using a realist evaluation approach, this paper aims to increase our understanding of how this type of service addresses patients’ dental anxiety in terms of its mechanisms and contextual factors. Interviews with TADA patients (*n* = 15) were analysed through a template analysis driven by context‐mechanism‐outcome heuristics. The analysis revealed that patients value a dental practitioner who provides a calm and holistic approach, positive judgements and predictability elements that lean towards a person‐centred care approach. Provided this, patients felt understood and cared for, their shame was reduced, self‐esteem emerged, and control was gained, which led to alleviation of dental anxiety. Therefore, our findings suggest that combining CBT with a person‐centred care approach helps alleviate patients’ dental anxiety. This provides insights into how dental services could be executed for these patients.

## INTRODUCTION

Many patients with a history of trauma or dental phobia struggle with attending routine dental examinations [1, 2, 3, 4]. One contributing factor is that the dental setting reminds patients of their trauma setting [5, 6]. This could be attributed to the dental examination being an invasive procedure that may include the administration of sharp objects into the mouth, being horizontally lowered, being alone in a room with a person of authority, the smell of the dental medication and the anticipation or experience of pain or judgement [4, 5]. These elements can trigger a physiological fight, flight, freeze, or faint response [4, 5, 6, 7, 8], with patients’ experiencing a sudden, overwhelming, and uncontrollable sensation of losing control and feeling threatened. This could result in patients losing their trust in the dental practitioner and the setting, which ultimately could lead to avoidance behaviour [9, 10, 11, 12, 13, 14, 15]. Avoiding dental services could be problematic because it could lead to deteriorated oral health and increased oral pain from infections, which would require more extensive and complex treatment procedures [16, 17, 18, 19]. The patient can then enter a recurring cycle of avoidance, dental neglect, enhanced awareness, and embarrassment over unmet needs and reduced psychosocial life and can eventually reinforce their initial dental fear and anxiety [20].

Cognitive behavioural therapy (CBT) may be one way to break the cycle of avoidance behaviour. CBT is a widely studied therapeutic approach initially intended to treat depression [21, 22], but some dentists also use it in their practice as an evidence‐based method to treat dental anxiety [16, 23]. Previous research involving dental practitioners who have used CBT on patients with dental phobia has shown a significant lower dental anxiety score, better dental service attendance and decreased decayed teeth counts after a 1‐year follow‐up [24, 25, 26]. A review by Wide Boman *et al*. [27] concluded that CBT is a promising therapy and often a therapy of choice for the treatment of patients with dental anxiety or phobias. A cognitive behavioural therapist assumes that the patient's cognition and thinking are disrupted, thus affecting dysfunctional emotion and behaviour [21]. In CBT, the goal is to unpack cognitive elements, such as thoughts, mental images, self‐talk, and core beliefs and subsequently alter them [21, 22]. Therefore, CBT may vary depending on the disorder or diagnostic symptoms displayed by the patient. For patients with dental anxiety disorders, CBT involves unpacking and testing patients’ catastrophic misinterpretations of the anxiety‐provoking setting or stimuli [14, 16]. This begins with the therapist gradually exposing the patient to feared objects or stimuli in the dental setting in a controlled fashion [14]. The therapist helps the patient through this process by setting the goals and tasks needed to achieve the dental treatment required. This process will be unique to the individual patient depending on their potential trauma background, anxiety triggers, and personal preferences, but the therapy centres on a hierarchical technique that habituates the patient towards the fear stimuli [14, 16].

Existing research has focused on assessing the efficacy of CBT outcomes for patients with dental phobia or anxiety [24, 25, 26]. However, less is known about the underlying mechanisms that explain why CBT is efficacious and which contextual elements trigger these mechanisms that lead to an outcome of either a successful or unsuccessful alleviation of dental anxiety. To address this knowledge gap, we take an in‐depth approach to explore patients’ experiences of an exemplar CBT service offered to phobic patients in a dental service in Norway, where dental practitioners themselves delivered the CBT. This study explores specifically *why (mechanisms) and under which circumstances (context) patients with a history of trauma or dental phobia who have undergone CBT as part of a specific service (torture, abuse, and dental anxiety [TADA]) experience alleviation of their dental anxiety*. In this way, our study contributes to the understanding of how dental practitioners deliver their services, particularly how using CBT alongside standard dental procedures may enhance this.

### Testing the theory of how the TADA service functions

In Norway, the TADA service was developed to address avoidance behaviour by catering to both patients’ anxiety and their oral health. The service first treats dental anxiety through CBT. When dental practitioners administer CBT, they do so as part of a collaborative and interprofessional team that includes psychologists. *Dental practitioner* is an umbrella term covering dentists, dental hygienists, and dental assistants. The division of labour is such that the dental practitioners administer the CBT through sessions of in‐vivo exposure therapy, and the psychologists assess patient service eligibility, oversee the dental practitioners’ delivery of CBT, and are in proximity to assist in the CBT intervention if needed [28, 29]. Patient eligibility for the service is assessed on criteria either outlined for dental phobia in the *Diagnostic and Statistical Manual IV* or in the patient's reported history of abuse or torture.

This paper explores patients’ experiences with CBT, as offered by the TADA service, by testing a theory of how the TADA service functions, as proposed by service deliverers and developers [28, 29]. This theory proposes that, from a professional perspective, the TADA service, through its bidimensional approach to providing dental anxiety treatment and dental restoration, works largely to reach a population that has otherwise been found to avoid dental services. As it is free of charge, the service is easy to access and, hence, meets individual needs. This is key to its functionality. From the deliverers’ perspective, the service tailors CBT to meet individual needs, and the dental practitioners adopted roles that enabled them to build patients’ trust, facilitate a safe space, and thereby grade the desensitisation regarding patients’ fear triggers. Adopting these roles requires dental practitioners to work in an institutional setting that provides sufficient time and additional skills, thus enhancing their interpersonal development and allowing them to adopt an appropriate communication style and therapeutic pace for each patient.

These findings [28, 29] reflect how developers and deliverers theorise the workings of the service; however, they were not informed by the recipients’ perspectives. The key to building on this understanding of the mechanisms that lead to ‘alleviated dental anxiety’ is to now include the patient's perspective [30]. According to Pawson and Tilley [30], different stakeholders are expected to have different knowledge about the service. Through service participation, patients are expected to be more sensitised to the mechanisms that lead to the outcome of ‘alleviated dental anxiety’ and can thus provide us with data on which resources lead to the change at the individual level. Therefore, this paper sought to refine this initial theorisation of the TADA service (a so‐called programme theory in realist terminology) [28, 29] by examining, comparing and contrasting this view of service developers and deliverers with the perspective of the TADA patients themselves. Researching this could provide insights into how dental practitioners could execute dental anxiety services for patients with dental phobia or a history of torture or abuse.

## MATERIAL AND METHODS

A realist evaluation approach offers a methodology to uncover the mechanisms and contexts at play [30]. It does so by theorising *what works for whom and under what circumstances*, *how and why* before collecting data to inform this initial theory [30]. By asking what is working *for whom* and *in what circumstances*, the realist approach identifies contextual elements. The philosophical underpinning of a realist evaluation assumes that specific contexts trigger a working mechanism, which answers *how and why* a service is working. Furthermore, it assumes that, when a context triggers a mechanism, this leads to an outcome that identifies *what* is working. By assuming that a context triggers a mechanism that leads to an outcome, this also implies that context, mechanisms and outcomes work as a contingency. Through this way of thinking, Pawson and Tilley [30] proposed that, in order to gain insight into the causal inferences of why something works and for whom and under which circumstances, the contextual factors, the mechanisms these trigger and the permutations in which these combine to achieve particular outcomes should be explored. The realist evaluation literature hence presents the formula of *context + mechanisms  =  outcomes* [30]. This formula has been subsequently modified by mechanisms being divided into the dyad of *resources and reasonings* [30, 31]. Reasonings refer to the explanations that people provide at a cognitive level for why they behave differently within a programme/service. Resources are those factors introduced into the context that then enable them to alter their reasoning. Thus, in realist evaluation language, the refined formula used in our analysis is represented by the following: *resource + context → reasoning = outcome* [30, 31].

The realist approach is a theory‐driven methodology and assumes that services are theory incarnate [30]. Therefore, the research steps involve identifying and formulating an initial programme theory, often set by service developers, which acts as a working hypothesis to be tested, often with service users. This initial programme theory navigates sampling and the method choices for data collection. This also means that, to fully understand how the TADA service works, for whom and under which circumstances, the realist approach allows us to build on the perspective of the service deliverers by comparing these with those put forward by the patients themselves.

The ontological assumption within realist evaluations is that mechanisms are often hidden and are thus difficult to measure through quantitative data method procedures [30, 32]. Therefore, a qualitative method was employed in the current study that consisted of in‐depth semi‐structured interviews with patients to explore their perspectives on why and under which circumstances CBT led to the outcome of ‘alleviated dental anxiety’. This choice of method allowed us to gain rich and descriptive data on the contexts and mechanisms, thus informing our initial programme theory.

### Interviews

As outlined in the initial programme theory, this study defined *alleviated dental anxiety* as the central outcome of the service. Therefore, the patients were purposively recruited based on having reached this outcome. In practical terms, this involved recruiting all patients within a specific region in Norway who had finished the CBT phase of the service. These patients were transitioning to the oral restoration team for continued oral treatment. Thus, the TADA service assessed them as having their anxiety alleviated [28].

This paper refers to all the patients enrolled in the TADA service generically as ‘TADA patients.’ We acknowledge that TADA patients are heterogeneous but consider their commonality as central: they all fear procedures related to the dental encounter and examination.

Data collection occurred from the end of December 2020 to the start of June 2021. Fifteen informants were recruited, and interviews were carried out by the lead author. The interviews were individual, as opposed to focus groups, based on the assumption that sensitive themes could emerge and group interviews could restrict information gathering.

An in‐depth semi‐structured interview guide consisting of six broad questions was formulated. Patients were asked about (1) their experience receiving CBT; (2) how their dental anxiety progressed, what led them to reach out to the TADA team and how this evolved into a treatment plan as part of the service pathway; (3) their experience during and post treatment, focusing on the positives and challenges and how they overcame challenges; (4) if and how treatment affected other areas of their life; (5) if and how the service could be different; and (6) their expectations and feelings on entering the dental restoration phase.

Causal follow‐up questions, such as ‘how did trust affect the treatment pathway,’ naturally arose and allowed the interviewer to better understand the themes that informed our initial programme theories. These predefined themes were time, communication, and pace (as contexts) and trust, safe space, and graded desensitisation (as mechanisms) and became naturally embedded as follow‐up questions for questions 1−3. Therefore, the interview style took a middle ground between a bottom‐up and top‐down approach that allowed the researcher to explore the existing themes already defined through the broader open‐ended questions presented in the paragraph above [33]. This technique allowed the possibility of new and emerging themes while limiting key concepts to the perspective of study and the initial programme theory.

### Analyses and data management

The duration of interviews averaged 42 min, with a maximum length of 81 min and a minimum of 22 min. Interviews were transcribed verbatim directly afterwards, and analyses ran concurrently with the data collection. A template analysis, including context‐mechanism‐outcome heuristics, was used to inform the initial programme theory [30, 32, 33]. The template analysis, proposed by King [33], is an iterative and flexible analytical process that encourages a focus on where the richest data lie. Therefore, it is an appropriate tool for applying a context‐mechanism‐outcome lens to focus on aspects of the data that are relevant to our research aims and can inform the initial programme theory developed in the early phases of the evaluation. The first analytical step was to become familiar with the collected data material and better understand the programme theory by reading interview transcripts. Second, preliminary codes, a priori themes, were outlined from our initial programme theory developed from interviews with professionals in the service. These thematic codes were identified as time, communication, and pace, which were placed under contexts, and trust, safe space, and graded desensitisation, which were placed under mechanisms [29].

**FIGURE 1:**
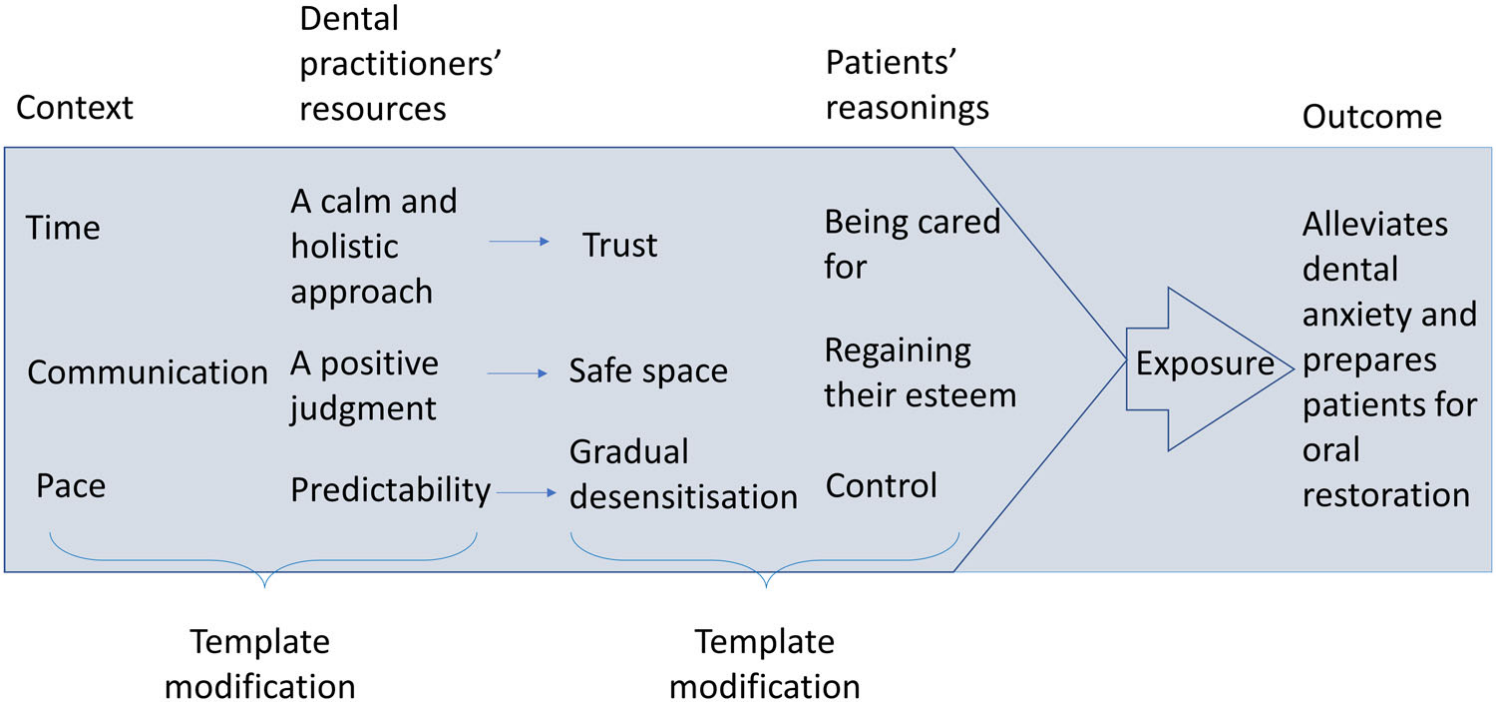
This figure represents how our a priori codes informed our context‐mechanism‐outcome configurations and followed a template modification. The text above the blue box refers to the realist context‐mechanism‐outcome heuristic tool used in the analyses, and the text below refers to which themes were modified to include the patient perspective in our programme theories

These themes worked as an initial coding template for the ongoing analyses, which was modified about halfway through the analyses, as the initial template was deemed inadequate for representing the patient's perspective. For transparency, the modification of the template is depicted in Figure 1. Modifying the template is part of the iterative process and is typical of both template analyses and realist evaluations [32, 33]. The modified template included themes of ‘a calm and holistic approach,’ ‘a positive judgement’ and ‘predictability,’ hierarchically structured under ‘dental resources.’ These showed causal links to themes placed under ‘patient reasonings,’ including ‘being cared for,’ ‘regaining their self‐esteem and devaluing shame’ and ‘control.’ These led to the outcome of ‘finishing CBT and alleviating dental anxiety.’ Thus, this modified template revealed the causal chain between context‐mechanism‐outcomes by having mechanisms split into dyads consisting of resources and reasonings, and the template was thus applied to the entire data set. After applying it to the entire data set, the template's wording was altered to ‘if…then’ statements, which, for the theory‐building process, further clarified which resources led to a change in the patient's reasoning. Realist evaluations often apply ‘if…then’ statements to evaluate casual links or as a translation between context‐mechanism‐outcome configurations, as they enhance the understanding of the interconnection between the themes and minimise the probability of simply cataloguing themes [30, 34, 35, 36, 37].

### Trustworthiness

The researcher kept a journal throughout the research process to reflect on background, subjectivity, and the potential impact on the data being collected and the analysis [38, 39]. Throughout the interviews, the researcher repeatedly paraphrased the information given by the participants to ensure that the interviewer and interviewee understood each other. The initial analysis by the first author was discussed and adjusted accordingly by the entire research team to establish the confirmability of our findings [40]. Quotes and the analytical procedure, as depicted in Figure 1, of the context‐mechanism‐outcome configurations have been added as part of the reporting strategy to gain credibility.

Lastly, the trustworthiness of the data interpretation could have been influenced by the previous data collection due to a defined initial programme theory. Nonetheless, from a realist perspective, Pawson and Tilley [30] suggested that this be a notion of perspectivism rather than a barrier to trustworthiness. As Pawson and Tilley [30] argued, an advantage of including multiple perspectives for refinement, such as those of deliverers from the initial programme theory and those of patients, is that validity is promoted, as the programme theories reflect various views from the actors involved. There are limitations to single perspectives; yet by accumulating these perspectives, the research can also inform the various views on why something works within specific services. Therefore, moving away from the perspective of whether something is working or not, one is moving toward a perspective that recognises the multiple perspectives and the human activity involved in whether interventions and services are successful.

### Ethical approval

The Norwegian Regional Ethics Committee (Project No: 134932) and the Norwegian National Centre of Research Data approved this project (Project No: 619754) and the study protocol. Patients were recruited after finishing their dental anxiety treatment; thus, assessed as *finalised* by the TADA service. The interview guide was explained beforehand to avoid potential participation implications, and psychological follow‐up by the TADA team was made available for the patients. None of the patients accepted this offer. Also, at the start of the interview, patients were encouraged to steer the conversation based on what they felt comfortable sharing and to discontinue whenever they would like.

## RESULTS

Eight males and seven females participated in this study. Seven participants had additional trauma histories resulting from either torture or abuse. Nine participants struggled with additional mental health challenges, such as posttraumatic depression, depression, and general anxiety. The period since the last dental visit varied; two had visited within the previous 2 years, seven within 2−5 years, two within 5−10 years, three within 15−20 years, and one between 20 and 25 years ago.

**FIGURE 2:**
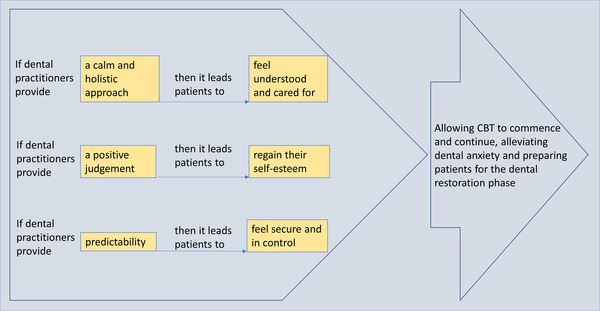
Converted template to include ‘if…then’ statements identifying how resources led to altered reasoning affecting the outcome of ‘alleviated dental anxiety.’ This illustrates the causal pathway between which resources led to which change of reasoning for the patients. The resources paired with the reasonings make up a dyad explaining the mechanisms involved that led to the outcome

In our description of the findings, we present a summary of the theories formulated by the patients in terms of a resources, context, reasoning, and outcome configuration shown in Table 1. These configurations are then followed by detailed descriptions of each refined theory presented under separate headings. The configurations in Table 1 serve as building blocks for programme theories that originated from the service developers’ perspectives, which can also be traced back to Figure 1. Figure 1 depicts the contextual themes and a priori codes from the analysis—‘time,’ ‘communication,’ and ‘pace’—which were modified to ‘a calm and holistic approach,’ ‘a positive judgement,’ and ‘predictability’ that fit under resources provided by the dental practitioners. Moreover, the a priori codes of ‘trust,’ ‘safe space,’ and ‘gradual desensitisation’ were modified under the patient's reasoning of ‘being cared for,’ ‘regaining their self‐esteem,’ and ‘control.’ The context, dyad of resources and reasoning, and outcome are labelled above the blue box in Figure 1 to indicate the realist heuristic tool used for analytical purposes. Building on Figure 1, we developed Figure 2 to enhance our retroductive theory‐building process and depict what patients described as dental practitioners’ resources that led to their change of reasoning. The text in the yellow boxes in Figure 2 first refers to the resources provided by the dental practitioner following the patients’ change of reasoning through the realist ‘if…then’ statements.

**TABLE 1:** Context‐mechanism‐outcome configurations building the programme theories

Dental practitioners' resource (Mechanism)	Context	Patients' reasoning (Mechanism)	Outcome
A holistic and calm approach	The patients generally fear dental practitioners and the tools involved in the dental setting and perceive the dental scenario and practitioners as rushed and incentivised by money, which overshadows their needs. The TADA setting deviates from this, as an institutional contextual layer to the service is time.	Patients' reasoning changes towards the setting; they feel understood and cared for in the TADA setting because they are met differently than envisioned, as they are treated with a holistic and calm approach.	Feeling understood cared for in a setting they feared allowed them to finish the dental anxiety treatment, which has alleviated their dental anxiety.
A positive judgement	TADA patients' avoidance behaviour and related shame hinder them in upholding a routine in dental examinations. The institutional TADA setting facilitates dental practitioners in developing interpersonal skills so that their communication becomes a tool to generate a safe space and provide patients with a positive judgement.	Patients ' shame is reduced by positively judging their oral status, and they start regaining their self‐esteem.	Regaining their esteem and reducing their shame has led to their approval of the service, which allowed them to continue and finish the dental anxiety treatment, which has alleviated their dental anxiety.
Predictability	CBT involves confronting feared objects/stimuli, which can trigger a fear response from the patients. In a context where the pace is matched with the patient's anxiety level, gradual desensitisation occurs.	Patients gain control because they become prepared and informed about exposure activities, length of activities, and stop signs during the session.	Being in control of the exposure sessions led to finishing the dental anxiety treatment, which has alleviated their dental anxiety.

*Abbreviations*: CBT, cognitive behavioural therapy; TADA, torture, abuse and dental anxiety.

### Programme Theory 1: A holistic and calm approach taken by the dentist leads patients to feel understood and cared for

The TADA patients unanimously explained that their dental anxiety was linked to the perception of dental practitioners as being rushed and seeing patients merely as objects and a source of income. They explained it as a ‘honk and drive’ setting that did not give dentists time to listen to their needs or build a practitioner–patient relationship. Thus, the patients were inclined to feel vulnerable and insecure about the anxiety responses that could arise in the dental setting.

The patients believed that the TADA setting deviated from this norm of general dental services. The institutional setting of the TADA service provides dental practitioners with more time, allowing them to build a trusting relationship with the patient through active listening and portraying sensitivity. This affected the patients’ view of the TADA dental setting, as their previous negative perception of the dental setting and practitioner was challenged. By being met by a practitioner who portrayed sensitivity towards their needs and actively listened to them, patients felt cared for and as if they met with understanding.

Patients thus agreed with the theory initially presented by the dental workers, but they expanded on this theory to suggest that they perceived the TADA dental practitioner as calm and holistic because, by understanding that their needs extend beyond the mouth, the dental practitioner saw the patient as a whole person. As Figure 2 depicts, this calm and holistic approach in a setting that had previously been associated with anxiety, insecurity and vulnerability allowed patients to feel understood and cared for. This was mentioned as a critical ingredient for the treatment pathway to both commence and continue, and this alleviated their anxiety. One patient noted,

‘I was met with understanding, even though I sat there crying my eyes out’ (Female, trauma, 2–5 years since last dental visit).

Other patients had similar experiences:

I wouldn't be here if she hadn't been a good dentist. But it depends on, what should I say, their personality, how they accept patients, how they treat you like a human being. (Male, dental phobia, 2–5 years since last dental visit)

Some dentists just get you down in the chair and open your mouth and say, ‘That's the problem’, fix it, and go out […] But if you have others who see you as a human being […] see your needs – what are you struggling with, also take that into account. (Male, dental phobia, 2−5 years since last dental visit)

### Programme Theory 2: Feeling that the dentist viewed them positively in the dental setting reduced patients’ feelings of shame and helped them regain their self‐esteem

TADA patients expressed feeling vigilant and self‐conscious regarding their oral status. Some patients said that, before the TADA treatment, they avoided social contact, covered their mouth from partners and avoided smiling to hide their oral status. Patients described their shame and fear of ‘revealing the mouth’ and said this nurtured their avoidance behaviour, and some had avoided dental services for decades. For these patients, high maintenance, including a vigorous tooth‐brushing regime, was described as a strategy to continue their avoidance behaviour and maintain specific control over their oral status. Patients with fewer years since their last dental visit explained that they had sought dental care for acute cases and pain relief. In these cases, the treatment procedures were often complex and painful, confirming the patient's fear of the dental setting.

Patients explained that it had taken much courage to seek help from the TADA service to change their avoidance behaviour, manage their anxiety and have their oral needs met due to their severe anxiety and shame associated with dental settings. The mere fact that the service addresses both psychological and dental needs played a role in service enrolment and following through with the service pathway. Following through with the service pathway was helped by the positive judgement that patients received from the dental practitioners.

Patient interviews elaborated on the dental practitioners’ initial programme theory, revealing that the institutional context of TADA facilitated dental practitioners in building a repertoire of communication skills that would provide a safe space for patients. Patients acknowledged the TADA dental practitioners’ skills in reducing the patients’ feelings of shame by providing them with a positive judgement of their oral status. Examples given by patients included remarks made by the dental practitioners, such as ‘I've seen worse’ or ‘This was not as bad as you explained it.’ As illustrated in Figure 2, providing patients with a positive judgement towards their oral status reduced their shame and enhanced their self‐esteem because they felt accepted. Patients described this as life‐changing and said that it motivated them to continue the treatment pathway and alleviated their anxiety:

‘We've got it; we've seen it all,’ she [the dental practitioner] says to me, so it doesn't matter. (Female, trauma, 15–20 years since last dental visit)

[…] the shame of not daring to go to the dentist because your teeth are broken and that things are not good. Just dining out and having to hide in the toilets with the toothpicks. So, it certainly has been a big life change for me – starting and doing this [the TADA treatment]. (Female, trauma, 15–20 years since last dental visit)

### Programme Theory 3: The predictability of the TADA sessions leads patients to feel a sense of control in this feared situation

Patients described a fear of losing control or experiencing anxiety responses, such as panic attacks, dissociating or fainting during exposure sessions. In the original programme theory, service deliverers explained the need to grade the CBT and match patients’ tolerance levels to avoid these anxiety responses.

Patients elaborated on this and included predictability as a necessary resource from the dental practitioners in the context. As exemplified in Figure 2, when dental practitioners provided patients with a predictable service, patients explained that they regained control, which diminished their fear and associated anxiety.

Patients explained that establishing predictability occurred both before and during the session. Establishing predictability during the session entailed dental practitioners reminding patients of their treatment plan, the exact length of time of the exposure and confrontation (e.g., 15 s with the oral injection needle inside the mouth) and their agreed stop sign for ending or pausing the session.

Predictability before the session varied for patients. The majority (all but one) expressed the importance of an introduction session held before the CBT began. In this session, patients are given information about the upcoming CBT sessions and the nature of the exposure activities that will soon commence. However, one patient, a female with a trauma background comorbid with other mental health challenges, deviated from this. This patient explained that knowing about the exposure activities before the CBT sessions began would only accelerate her fear and result in her cancelling the session. Thus, for this specific patient, it was essential to establish predictability of exposure activities only at the onset of the CBT sessions instead of at the induction phase.

In either case, patients expressed that, when dental practitioners established predictability, it allowed them to gain control in this setting, as one patient noted

‘because you have 100% confidence in what is happening, which has allowed you to tune in to it, and a 100% specific thing to dread, as opposed to dreading a sea of things, and that certainty.’ (Male, dental phobia, 15–20 years since dental visit)

One stated:

‘Being aware of what's going to happen today, that was important. It calmed me down a lot. More than just being put in a chair and not knowing anything.’ (Male, dental phobia, 2–5 years since last dental visit)

Patients described that, by having control in a setting that they previously feared, they now felt able to continue the treatment pathway and have their anxiety alleviated:

They make it so that you don't worry unnecessarily about a treatment that you might think is getting worse and more serious than it actually will, because you know everything is predictable. (Male, dental phobia, 15–20 years since dental visit)

## DISCUSSION

In this study, patients with dental phobia or trauma history identified essential resources for them in the TADA therapeutic setting. Patients reported that their dental anxiety was alleviated because dental practitioners provided them with a calm and holistic approach, positive judgements and predictability. When dental practitioners provided these resources within the therapeutic context, patients reported feeling more controlled in the setting. They felt cared for and understood, their shame was reduced, and they gained self‐esteem. This led to patients’ commencing and continuing the CBT, alleviation of their dental anxiety, and preparing for their dental restoration. The causal pathway between dental practitioners’ resources and patients’ reasonings is illustrated in Figure 2. One of these resources, and the first identified in our context‐mechanism‐outcome configurations, uncovered that patients’ dental anxiety was alleviated in the TADA setting because patients felt that the dental practitioners provided a calm and holistic approach that made them feel understood and cared for. Research on vulnerable patients’ perceptions of care services has shown that a feeling of being understood and cared for is vital for keeping dignity, easing suffering, and contributing to clinical decision‐making [41, 42, 43]. Furthermore, research has found that creating interpersonal relationships can affect treatment outcomes, suggesting that the practitioner–patient relationship goes beyond the type of treatment provided [44].

A further resource was identified by patients, as outlined in our second context‐mechanism‐outcome configuration: patients felt that practitioners provided a positive judgement that reduced their feelings of shame and led them to regain their self‐esteem. Research has shown that patients with dental anxiety tend to score low in self‐esteem, often resulting in avoidance of social activities and relationships due to shame about their dental appearance [45, 46]. Reducing feelings of shame and thereby enhancing self‐esteem means there is potential for improving patients’ well‐being [6].

The last resource noted by patients, which was identified in the final context‐mechanism‐outcome configuration, shows that patients felt that dental anxiety was alleviated in the TADA setting because dental practitioners provided them with predictability, which led them to gain control. Control in this setting refers to the ability to affect the outcomes in the situation, potentially avoiding or limiting adverse events within the given scenario. Previous research has also identified control as a mechanism that affects dental anxiety [3, 47], and research on control within the psychology field has suggested that control promotes coping with stressors [48, 49]. Our findings contribute to existing research, as our analyses display specifically how the dental practitioners provided predictability – allowing patients to gain control. This involved reminding the patients of the treatment plan, activities’ duration and the agreed‐upon stop signs that would end or pause the session. Thompson [49] described this type of predictability as informational, as it allows patients to gain control because they become prepared and informed about how to affect the potential adversity of outcomes.

The patients’ emphasis on *how* the dental practitioners delivered the therapy rather than the specific components or strategies of the CBT itself is interesting and surprising, as the focus of the training for dental practitioners in the TADA service was to deliver CBT. We, therefore, argue that the three identified resources give rise to a more person‐centred care approach [50, 51], which adds complexity to the therapeutic CBT interventions. The person‐centred care approach, which branches from humanistic psychology, is defined as ‘providing care that is respectful of and responsive to individual patient preferences, needs, and values and ensuring that patient values guide all clinical decisions’ [52]. A person‐centred care approach deviates from CBT in that it is less focused on the instrumental tools for altering the cognition and habituation towards fear stimuli [14]. Instead, the person‐centred care approach focuses on the patient's ways of becoming an active agent, thus creating a positive atmosphere where patients are met with respect, empathy and understanding and are actively listened to Mills *et al*. (2013) [53]. Hence, when dental practitioners explore the patient's perspective, it allows them to build a practitioner–patient relationship and see the patient holistically.

The findings from this study add to our findings from the first phase of the TADA evaluation [29], where our initial theory described *pace*, *time* and *communication* as important aspects of the service's success, as seen from the service deliverers’ perspective. By testing this initial theory after the patients have received the service, we uncovered the specific *ingredients* of the active mechanisms, namely, which resources were essential for reaching the service's end goal of alleviating their dental anxiety. Thus, a major strength of this study is its perspective on evaluating the TADA service based on the patients’ experiences. This has provided us with valuable insight and a detailed description of what they found important in reaching the outcome of ‘alleviated dental anxiety’ rather than a clinical measurement outcome.

A notable limitation in this study is that the patients participating in this study were likely to be highly motivated, as they had ended their avoidance behaviour, sought contact and finished the service treatment pathway. Furthermore, our ethical decision to only interview patients who had finished the service pathway also brought about the limitation of collecting data from patients for whom the service has been a success. This means that, while we better understand what works for whom and under which circumstances, we have less of an idea of what does *not* work for whom under which circumstances.

Our findings hold implications for how dental services can be structured to deliver therapeutic interventions. Scambler and Asimakopoulou [50] proposed a model of how to incorporate patient‐centred care within dentistry that consists of foundational components that involve (1) exploring how the patient's disease affects the patient, (2) seeing the patient as a whole, (3) providing ethos that is compassionate and empathetic, and (4) reaching a mutual agreement on treatment goals. Our results support this model from a patient perspective and suggest that this model could be effective for vulnerable patients with dental anxiety. Therefore, we suggest that dental services should implement a person‐centred care approach as part of CBT when designing future services for patients with a history of trauma or dental phobia. This is further supported by research elsewhere showing that a person‐centred care approach increases patient satisfaction, reduces service utilisation, and becomes more efficient in terms of time spent during consultations, thus proving to be cost‐saving [54, 55, 56, 57].

In conclusion, while dentistry traditionally is seen as a profession that focuses on technical procedures, an approach in which the practitioner sees the patient before the teeth is valuable for vulnerable groups. As depicted in Figure 2, our findings describe that, if dental practitioners provide vulnerable patients with a calm and holistic approach, a positive judgement and predictability, these patients will feel understood and cared for, regain their self‐esteem, and feel secure and in control. The patients identified this as leading to their outcome of ‘alleviated dental anxiety.’ The findings build on our initial programme theory set by the TADA service deliverers that indicate pace, time and communication as important aspects in alleviating anxiety for this patient group [29] yet uncovered specific resources provided by the dental practitioner that lean more towards a person‐centred care approach. These findings also suggest that the dental practitioner approach can mediate therapeutic outcomes. However, it remains unclear how a person‐centred care approach and CBT complement each other in the clinical setting. Future research is required to explore dental practitioners’ views on the impact of CBT training. It should explore whether CBT training, as reported in our study by the patients themselves, does indeed expand the dentists’ vision beyond just the patient's mouth and whether, from the dentists’ perspective, time, and the removal of performance‐based measurement impact the dentists’ perspective on the patient–practitioner therapeutic relationship.

## CONFLICTS OF INTEREST

Units of the TADA service are located at the Oral Health Centre of Expertise, Rogaland, Norway.

## AUTHOR CONTRIBUTIONS

**Conceptualisation**: Emilie Bryne, Sarah Hean, Kjersti Berge Evensen, Vibeke Hervik Bull; **Methodology**: Emilie Bryne, Sarah Hean; **Validation**: Emilie Bryne, Kjersti Evensen; **Formal Analysis** and **Investigation**: Emilie Bryne; **Data Curation**: Emilie Bryne, Sarah Hean, Kjersti Berge Evensen, Vibeke Hervik Bull**; Writing – Original Draft**: Emilie Bryne; **Writing – Review & Editing**: Emilie Bryne, Sarah Hean, Kjersti Berge Evensen, Vibeke Hervik Bull; **Visualisation**: Emilie Bryne; **Supervision**: Sarah Hean, Kjersti Berge Evensen, Vibeke Hervik Bull.

## References

[1] Milgrom P , Vignehsa H , Weinstein P . Adolescent dental fear and control: prevalence and theoretical implications. Behav Res Ther. 1992;30:367–73. 1616471 10.1016/0005-7967(92)90048-l

[2] Abrahamsson KH , Berggren U , Hakeberg M , Carlsson SG . Phobic avoidance and regular dental care in fearful dental patients: a comparative study. Acta Odontol Scand. 2001;59:273–9. 11680645 10.1080/000163501750541129

[3] Abrahamsson KH , Berggren U , Hallberg L , Carlsson SG . Dental phobic patients' view of dental anxiety and experiences in dental care: a qualitative study. Scand J Caring Sci. 2002;16:188–96. 12000673 10.1046/j.1471-6712.2002.00083.x

[4] Larijani HH , Guggisberg M . Improving clinical practice: what dentists need to know about the association between dental fear and a history of sexual violence victimisation. Int J Dent. 2015;452814. 10.1155/2015/452814. 25663839 PMC4309219

[5] Leeners B , Stiller R , Block E , Görres G , Imthurn B , Rath W . Consequences of childhood sexual abuse experiences on dental care. J Psychosom Res. 2007;62:581–8. 17467413 10.1016/j.jpsychores.2006.11.009

[6] Cohen SM , Fiske J , Newton JT . The impact of dental anxiety on daily living. Br Dent J. 2000;189:385–90. 11081950 10.1038/sj.bdj.4800777

[7] Bracha HS . Freeze, flight, fight, fright, faint: adaptationist perspectives on the acute stress response spectrum. CNS Spectr. 2004;9:679–85. 15337864 10.1017/s1092852900001954

[8] Moore R , Brødsgaard I , Rosenberg N . The contribution of embarrassment to phobic dental anxiety: a qualitative research study. BMC Psychiatry. 2004;4:10. 10.1186/1471-244X-4-10 15096278 PMC411042

[9] Keller SM , Zoellner LA , Feeny NC . Understanding factors associated with early therapeutic alliance in PTSD treatment: adherence, childhood sexual abuse history, and social support. J Consult Clin Psychol. 2010;78:974–9. 20873895 10.1037/a0020758 PMC3244206

[10] Fredriksen TV , Søftestad S , Kranstad V , Willumsen T . Preparing for attack and recovering from battle: understanding child sexual abuse survivors' experiences of dental treatment. Community Dent Oral Epidemiol. 2020;48:317–27. 32436226 10.1111/cdoe.12536

[11] Willumsen T . The impact of childhood sexual abuse on dental fear. Community Dent Oral Epidemiol. 2004;32:73–9. 14961843 10.1111/j.1600-0528.2004.00120.x

[12] Wolf E , McCarthy E , Priebe G . Dental care – an emotional and physical challenge for the sexually abused. Eur J Oral Sci. 2020;128:317–24. 33856710 10.1111/eos.12720

[13] Kranstad V , Søftestad S , Fredriksen TV , Willumsen T . Being considerate every step of the way: a qualitative study analysing trauma‐sensitive dental treatment for childhood sexual abuse survivors. Eur J Oral Sci. 2019;127:539–46. 31731327 10.1111/eos.12661

[14] Abramowitz JS , Deacon BJ , Whiteside SPH . Exposure Therapy for Anxiety. 2nd ed. New York: Guilford Press; 2019.

[15] Ehring T , Welboren R , Morina N , Wicherts JM , Freitag J , Emmelkamp PMG . Meta‐analysis of psychological treatments for posttraumatic stress disorder in adult survivors of childhood abuse. Clin Psychol Rev. 2014;34:645–57. 25455628 10.1016/j.cpr.2014.10.004

[16] Öst L‐G , Skaret E . Cognitive Behavioral Therapy for Dental Phobia and Anxiety. West Sussex, UK: John Wiley & Sons; 2013.

[17] Armfield JM , Stewart JF , Spencer AJ . The vicious cycle of dental fear: exploring the interplay between oral health, service utilization and dental fear. BMC Oral Health. 2007;7:1. 10.1186/1472-6831-7-1 17222356 PMC1784087

[18] Appukuttan DP . Strategies to manage patients with dental anxiety and dental phobia: literature review. Clin Cosmet Investig Dent. 2016;8:35–50. 10.2147/CCIDE.S63626 PMC4790493 27022303

[19] Zinke A , Hannig C , Berth H . Comparing oral health in patients with different levels of dental anxiety. Head Face Med. 2018;14:25. 10.1186/s13005-018-0182-4 30458845 PMC6247764

[20] Berggren U , Meynert G . Dental fear and avoidance: a study of etiology, consequences and treatment. J Am Dent Assoc. 1984;109:247–51. 6590605 10.14219/jada.archive.1984.0328

[21] Beck AT . Thinking and depression: II. Theory and therapy. Arch Gen Psychiatry. 1964;10:561–71. 14159256 10.1001/archpsyc.1964.01720240015003

[22] Beck AT . Thinking and depression: I. Idiosyncratic content and cognitive distortions. Arch Gen Psychiatry. 1963;9:324–33. 14045261 10.1001/archpsyc.1963.01720160014002

[23] Davis III TE , May A , Whiting SE . Evidence‐based treatment of anxiety and phobia in children and adolescents: current status and effects on the emotional response. Clin Psychol Rev. 2011;31:592–602. 21482318 10.1016/j.cpr.2011.01.001

[24] Vika M , Skaret E , Raadal M , Öst L‐G , Kvale G . One‐ vs. five‐session treatment of intra‐oral injection phobia: a randomized clinical study. Eur J Oral Sci. 2009;117:279–85. 19583756 10.1111/j.1600-0722.2009.00628.x

[25] Lillehaug Agdal M , Raadal M , Skaret E , Kvale G . Oral health and oral treatment needs in patients fulfilling the DSM‐IV criteria for dental phobia: possible influence on the outcome of cognitive behavioral therapy. Acta Odontol Scand. 2008;66:1–6. 18320411 10.1080/00016350701793714

[26] Haukebø K , Skaret E , Öst L‐G , Raadal M , Berg E , Sundberg H , et al. One‐ vs. five‐session treatment of dental phobia: a randomized controlled study. J Behav Ther Exp Psychiatry. 2008;39:381–90. 18005932 10.1016/j.jbtep.2007.09.006

[27] Wide Boman U , Carlsson V , Westin M , Hakeberg M . Psychological treatment of dental anxiety among adults: a systematic review. Eur J Oral Sci. 2013;121:225–34. 23659254 10.1111/eos.12032

[28] Bryne E , Hean S , Evensen KB , Bull VH . Exploring the contexts, mechanisms and outcomes of a dental anxiety service in Norway: a Realist evaluation. Research Square. Preprint. 2021. 10.21203/rs.3.rs-279468/v1 PMC9026053 35459239

[29] Bryne E , Hean S , Evensen K , Bull V . More than just a dental practitioner. Eur J Oral Sci. 2021. 10.1111/eos.12820 34448277

[30] Pawson R , Tilley N . Realistic Evaluation. London: Sage; 1997.

[31] Dalkin SM , Greenhalgh J , Jones D , Cunningham B , Lhussier M . What's in a mechanism? Development of a key concept in realist evaluation. Implement Sci. 2015;10:49. 10.1186/s13012-015-0237-x 25885787 PMC4408605

[32] Emmel N , Greenhalgh J , Manzano A , Monaghan M , Dalkin S . Doing Realist Research. London: Sage; 2018.

[33] King N . Doing template analysis. In: Symon G , Cassell C , editors. Qualitative Organizational Research. London: Sage; 2012. pp. 426–50

[34] Ebenso B , Manzano A , Uzochukwu B , Etiaba E , Huss R , Ensor T , et al. Dealing with context in logic model development: reflections from a realist evaluation of a community health worker programme in Nigeria. Eval Program Plann. 2019;73:97–110. 30578941 10.1016/j.evalprogplan.2018.12.002 PMC6403102

[35] Mukumbang FC , Marchal B , Van Belle S , van Wyk B . A realist approach to eliciting the initial programme theory of the antiretroviral treatment adherence club intervention in the Western Cape Province, South Africa. BMC Med Res Methodol. 2018. 10.1186/s12874-018-0503-0 PMC5970495 29801467

[36] Leeuw FL . Reconstructing program theories: methods available and problems to be solved. Am J Eval. 2003;24:5–20.

[37] Jagosh J . Retroductive theorizing in Pawson and Tilley's applied scientific realism. J Crit Realism. 2020;19:121–30.

[38] Patton MQ . Part 3: analysis, interpretation and reporting. In: Qualitative Research and Evaluation Methods, 4th ed. California: Sage; 2015. p. 652–743.

[39] Darawsheh W . Reflexivity in research: promoting rigour, reliability and validity in qualitative research. Int J Ther Rehabil. 2014;21:560–8.

[40] Polit DF , Beck CT . Nursing Research: Principles and Methods. Philidelphia: Lippincott Williams & Wilkins; 2004. p. 724.

[41] Elwyn G , Lloyd A , May C , van der Weijden T , Stiggelbout A , Edwards A , et al. Collaborative deliberation: a model for patient care. Patient Educ Couns. 2014;97:158–64. 25175366 10.1016/j.pec.2014.07.027

[42] Davis E , Tamayo A , Fernandez A . ‘Because somebody cared about me. That's how it changed things’: homeless, chronically ill patients’ perspectives on case management. PloS One. 2012;7(9):e45980. 10.1371/journal.pone.0045980 23029350 PMC3461032

[43] Epstein RM , Gramling RE . What is shared in shared decision making? Complex decisions when the evidence is unclear. Med Care Res Rev. 2013;70:94S–112S. 23035055 10.1177/1077558712459216

[44] Levant R . Report of the 2005 Presidential Task Force on Evidence‐based Practice. Washington, DC: American Psychological Association; 2005.

[45] Schuurs AH , Duivenvoorden HJ , Makkes PC , van Velzen SKT , Verhage F . Personality traits of patients suffering extreme dental anxiety. Community Dent Oral Epidemiol. 1988;16:38–41. 3422617 10.1111/j.1600-0528.1988.tb00552.x

[46] Berggren U . Psychosocial effects associated with dental fear in adult dental patients with avoidance behaviours. Psychol Health. 1993;8:185–96.

[47] Scandurra C , Gasparro R , Dolce P , Bochicchio V , Muzii B , Sammartino G , et al. The role of cognitive and non‐cognitive factors in dental anxiety: a mediation model. Eur J Oral Sci. 2021;e12793. 10.1111/eos.12793 33945646 PMC8453836

[48] Glass DC , Singer JE , Friedman LN . Psychic cost of adaptation to an environmental stressor. J Pers Soc Psychol. 1969;12:200–10. 5803831 10.1037/h0027629

[49] Thompson SC . Will it hurt less if I can control it? A complex answer to a simple question. Psychol Bull. 1981;90:89–101. 7267899

[50] Scambler S , Asimakopoulou K . A model of patient‐centred care – turning good care into patient‐centred care. Br Dent J. 2014;217:225–8. 25213518 10.1038/sj.bdj.2014.755

[51] Scambler S , Delgado M , Asimakopoulou K . Defining patient‐centred care in dentistry? A systematic review of the dental literature. Br Dent J. 2016;221:477–84. 27767160 10.1038/sj.bdj.2016.777

[52] Institute of Medicine Report: Crossing the Quality Chasm: A New Health Care System for the 21st Century. Washington, DC; 2001. Report No.: 1527–1544 Contract No.: 3.

[53] Mills I , Frost J , Moles D , Kay E . Patient‐centred care in general dental practice: sound sense or soundbite? Br Dent J. 2013;215:81–5. 23887534 10.1038/sj.bdj.2013.684

[54] Stewart M , Ryan BL , Bodea C . Is patient‐centred care associated with lower diagnostic costs? Healthc Policy. 2011;6:27–31. PMC3107114 22548095

[55] Bensing JM , Roter DL , Hulsman RL . Communication patterns of primary care physicians in the United States and the Netherlands. J Gen Intern Med. 2003;18:335–42. 12795731 10.1046/j.1525-1497.2003.10735.x PMC1494863

[56] Bertakis KD , Azari R . Patient‐centered care is associated with decreased health care utilization. J Am Board Fam Med. 2011;24:229–39. 21551394 10.3122/jabfm.2011.03.100170

[57] Bertakis KD , Azari R . Determinants and outcomes of patient‐centered care. Patient Educ Couns. 2011;85:46–52. 20801601 10.1016/j.pec.2010.08.001

